# Biodegradation of the High Explosive Hexanitrohexaazaiso-wurtzitane (CL-20)

**DOI:** 10.3390/ijerph6041371

**Published:** 2009-04-09

**Authors:** Pelin Karakaya, Christos Christodoulatos, Agamemnon Koutsospyros, Wendy Balas, Steve Nicolich, Mohammed Sidhoum

**Affiliations:** 1 Langan Engineering and Environmental Services, Elmwood Park, NJ 07407, USA; 2 Stevens Institute of Technology, Hoboken, NJ 07030, USA; 3 Mechanical, Civil and Environmental Engineering Department, University of New Haven, West Haven, CT 06516, USA; 4 US Army TACOM-ARDEC, Energetics & Warheads Division, Picatinny Arsenal, NJ 07806, USA; 5 PMK Group, Inc., ERCP Division, Farmingdale, NJ 07727, USA

**Keywords:** Hexanitrohexaazaisowurtzitane, CL-20, biodegradation, activated sludge, *Phanerochaete chrysosporium*, logistic growth model, mineralization

## Abstract

The aerobic biodegradability of the high explosive CL-20 by activated sludge and the white rot fungus *Phanerochaete chrysosporium* has been investigated. Although activated sludge is not effective in degrading CL-20 directly, it can mineralize the alkaline hydrolysis products. *Phanerochaete chrysosporium* degrades CL-20 in the presence of supplementary carbon and nitrogen sources. Biodegradation studies were conducted using various nutrient media under diverse conditions. Variables included the CL-20 concentration; levels of carbon (as glycerol) and ammonium sulfate and yeast extract as sources of nitrogen. Cultures that received CL-20 at the time of inoculation transformed CL-20 completely under all nutrient conditions studied. When CL-20 was added to pre-grown cultures, degradation was limited. The extent of mineralization was monitored by the ^14^CO_2_ time evolution; up to 51% mineralization was achieved when the fungus was incubated with [^14^C]-CL-20. The kinetics of CL-20 biodegradation by *Phanerochaete chrysosporium* follows the logistic kinetic growth model.

## 1. Introduction

The high-energy polycyclic nitramine (2,4,6,8,10,12-hexanitro-2,4,6,8,10,12-hexaazaisowurtzitane), commonly known as CL-20, is a synthesized energetic compound [1]. Due to its superior explosive properties, it is anticipated to replace conventional energetic materials in the near future [2,3]. However, information on environmental fate and health impacts, as well as potential remedial schemes, is rather limited.

**Figure 1.**
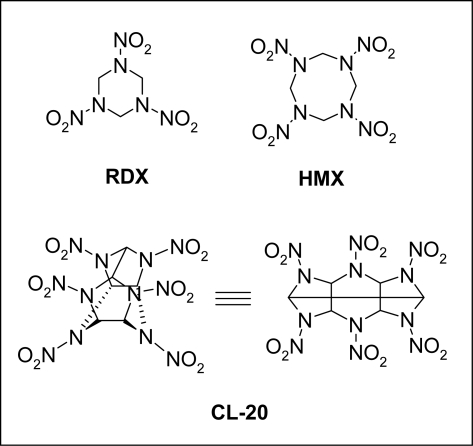
Molecular structures of RDX, HMX and CL-20.

The basic structure of CL-20 consists of a rigid isowurtzitane cage with a nitro group attached to each of the six bridging nitrogen atoms. Structurally, as shown in Figure 1, it resembles the conventional monocyclic explosives cyclotetramethylenetetranitramine (HMX) and cyclotrimethylenetrinitramine (RDX). Therefore, it is likely that CL-20 may present similar environmental challenges to HMX and RDX [4]. Preliminary studies have reported that CL-20 exhibits similar ecotoxicological potency as HMX and RDX [5]. CL-20 is not toxic to marine bacteria *Vibrio fischeri*, freshwater green algae *Selenastrum capricornutum*, terrestrial higher plants and indigenous soil microorganisms [5]. However, it is highly toxic to the soil invertebrates such as the earthworm *Eisenia Andrei* [6]. Shen *et al.* [7] reported that RDX is recalcitrant to aerobic sludge. On the other hand, HMX was amenable to biodegradation using activated sludge in bench and pilot scale studies [8].

Few studies have addressed the microbial degradation of CL-20 to date. Fournier *et al.* [9] reported that the white rot fungi *Phanerochaete chrysosporium* and *Irpex lacteus* can decompose CL-20 under aerobic conditions. Soil bacteria such as the aerobic *Agrobacterium sp*. strain JS71 [10] and the anaerobic denitrifier *Pseudomonas sp*. strain FA1 [11] were capable of degrading CL-20 when used as the sole nitrogen source. *Clostridium sp*. Strain EDB2 could also transform CL-20 anaerobically [12]. According to Trott *et al.* [10], CL-20 is readily biodegradable in soil environments; however, it is not clear whether aerobic or anaerobic soil conditions favor biodegradation. It has been suggested that the nitramine is biotransformed via a *N*-denitration mechanism, as confirmed by the detection of nitrite ions, nitro-labeled nitrous oxide, and doubly denitrated CL-20 intermediates during treatment with nitroreductase [13]. Nitro-labeled nitrous oxide and doubly denitrated CL-20 intermediates have also been detected during incubation with *Phanerochaete chrysosporium* [9]. Although the exact mechanism of action of the white rot fungi is not known, it has been postulated that the organisms are capable of decomposing low-solubility chemicals by producing highly oxidative extracellular enzymes. Moreover, the nonspecific enzymatic system of these fungi allows them to degrade a range of persistent nitrated explosives including RDX and HMX [14–17], as well as many other organic compounds. Enzymes essential for the biodegradation of chemicals are usually secreted by *Phanerochaete chrysosporium* in response to nitrogen or carbon starvation, as a part of its ligninolytic system, which consists of lignin peroxidases (LiP) and manganese-dependent peroxidases (MnP), along with hydrogen peroxide generating oxidases [18].

Although several studies have provided evidence of aerobic biotransformation of CL-20, to the best of our knowledge, data on degradability of CL-20 or its metabolites with mixed activated sludge cultures have not been reported. For this purpose, microcosm studies were conducted using different growth media. Biotransformation of the alkaline hydrolysis by-products of CL-20 has also been investigated. It was previously shown that alkaline hydrolysis is an effective method of converting CL-20 into smaller and soluble products (hydrolysates) [19,20]. The nature and quantity of these products remain unknown. Some studies have indicated that a synthetic mixture of hydrolysates (acetate, formate, formaldehyde and nitrite) resulting from the alkaline treatment of RDX can be further treated biologically [21,22].

The objectives of the present study are: a) to assess the aerobic biodegradability of CL-20 with activated sludge and *Phanerochaete chrysosporium*; b) to evaluate the potential mineralization of CL-20 hydrolysates using uniformly labeled [^14^C]-CL-20 and aerobic activated sludge; c) to explore the kinetics and extent of transformation of CL-20 by *Phanerochaete chrysosporium* in growing and pre-grown cultures, at different nutrient media compositions. This information is important for designing remedial systems for the treatment of potentially CL-20 polluted media.

## 2. Experimental Section

### 2.1. Reagents and Chemicals

CL-20, manufactured by A.T.K. Thiokol Propulsion (Brigham City, UT), was supplied by Picatinny Arsenal, NJ. CL-20 had a purity greater than 99% (determined by HPLC) and an ɛ-polymorph content greater than 98% (determined by Fourier Transform Infrared Spectroscopy), with an average particle size of 2 μm and a uniformity coefficient of 1.47. The solvents used, acetonitrile and acetone, were HPLC grade (Aldrich Chemical Co., Milwaukee, WI.). The uniformly labeled [^14^C]-CL-20, supplied by A.T.K. Thiokol Propulsion (Brigham City, UT), had a specific radioactivity of 0.73 μCi/g [^14^C]. Glucose and other reagents were obtained from Sigma-Aldrich (St. Louis, MO).

### 2.2. Analytical Methods

CL-20 was analyzed with a reverse-phase HPLC system (Varian Inc., Walnut Creek, CA) equipped with a photodiode array detector. A Symmetry Shield^™^ RP-18 (3.9 mm × 150 mm) 5 μm column (Waters^®^, Milford, MA) was used. The U.S. EPA Standard Method 8330, originally developed for the analysis of nitroaromatic and nitramine explosives was used [23]. The analysis conditions have been described in detail previously [19]. The detection limit for CL-20 was 0.05 mg/L. Glycerol concentration was determined using an enzymatic assay kit (R-Biopharm, SouthMarshall, MI). The absorbance was measured at a wavelength of 340 nm by UV spectrophotometry. Ammonium ion concentration was determined using a ThermoOrion 920A pH/ISE meter. The ^14^CO_2_ radioactivity was measured using a Tricarb 2900TR liquid scintillation analyzer (Pelkin Elmer, Shelton, CT).

### 2.3. Direct Treatment of CL-20 with Activated Sludge

Batch experiments were performed in 60-ml glass vials with activated sludge seed from the Bergen County wastewater treatment plant (Bergen County Utility Authority, Little Ferry, NJ). Four different growth-media were used: a) one set of vials was supplemented with glucose (2 g/L) as a carbon source, b) a second set was supplemented with ammonium sulfate as a nitrogen source (0.5 g/L), c) a third set contained both and d) the fourth set did not contain any supplement. The growth medium used consisted of: 8.5 mg/L KH_2_PO_4_, 21.75 mg/L K_2_HPO_4_, 33.4 mg/L Na_2_HPO_4_· 7H_2_O, 1.7 mg/L NH_4_Cl, 22.5 mg/L MgSO_4_, 27.5 mg/L CaCl_2_, and 0.25 mg/L FeCl_3_· 6H_2_O; pH 7.0. Microcosms were prepared by inoculating 60-mL glass vials containing 5 mL of growth medium, with activated sludge. 50 μL from a concentrated stock solution (10 g/L CL-20 dissolved in acetone) were added to each vial to attain a final concentration of 100 mg/L. Control microcosms containing autoclaved growth media without inoculum, were set up to assess potential abiotic transformations. All vials were incubated at 25 °C with agitation at 150 rpm in a constant temperature incubator. CL-20 concentration was monitored by extracting the remaining CL-20 in the aqueous cultures using ethyl acetate as a solvent instead of acetonitrile. The reason behind this substitution was the observed instability of CL-20 in acetonitrile-water mixtures at neutral pHs that has been reported in the literature [24]. CL-20 extracted with 5 mL of ethyl acetate was added into the sacrificed vial. The vial was then sealed and tumbled 50 times. The top ethyl acetate phase was transferred into a clean vial. The extraction procedure was repeated three times and the combined recovered ethyl acetate extract was evaporated at room temperature. The resulting dry residue, dissolved in 5 mL of acetonitrile, was subsequently analyzed by HPLC.

### 2.4. Treatment of Radiolabeled [^14^C]-CL-20 Hydrolysates with Activated Sludge

Batch mineralization experiments were conducted to assess the biodegradability of CL-20 hydrolysates in aerobic activated sludge cultures, at 30 °C in a constant temperature incubator with shaking (150 rpm). [^14^C]-CL-20 was subjected to complete alkaline hydrolysis in six 125-ml culture flasks. Each flask containing 50 mg/L [^14^C]-CL-20 solution (73.0 nCi) was treated with 0.1 M NaOH for 2 hours and afterwards neutralized using 1 mL of a 5 M HCl stock solution. 0.5 mL of concentrated (x100) mineral medium was then added into each flask. Next, the flasks were inoculated by adding 1 mL of aerobic sludge each to obtain a 1:50 inoculum ratio (by volume). Three of these flasks were used as sterile controls by adding HgCl_2_ (1 g/L) to inhibit microbial activity. Three positive controls with [^14^C]-glucose (50 mg/L, 73.3 nCi), instead of the hydrolyzed [^14^C]-CL-20 solution, were also prepared. Next, a sterile glass tube containing 4 mL of 0.5 N NaOH was placed in each flask to trap the ^14^CO_2_ generated from the mineralization of the ^14^C-labeled substrate. All flasks were sealed with PTFE Mininert® screw-cap valves to prevent the loss of ^14^CO_2_ during incubation. The trapped ^14^CO_2_ was monitored by periodically withdrawing the NaOH solution from the test tube and measuring the radioactivity using liquid scintillation counting. After sampling, 60 mL of the headspace air were replaced with the same volume of filtered (0.2 μm) fresh air to maintain aerobic conditions, and fresh NaOH solution was added through the screw-cap valve.

### 2.5. Biotransformation of CL-20 by Phanerochaete Chrysosporium

The fungal strain *Phanerochaete chrysosporium* (ATCC-24725) used was maintained on malt agar slants (20 g/L agar, 20 g/L malt extract, and 1 g/L yeast extract) at 37 °C. A spore suspension was prepared from the slants and stored at 4 °C. Batch biodegradation experiments were conducted at initial CL-20 concentrations below the aqueous solubility limit (1, 3 and 7 mg/L) and above solubility (100 and 500 mg/L). The aqueous solubility of CL-20 at 39 °C is 8·10 mg/L [19]. The fungal cultures were incubated aerobically in the dark and maintained at 39 ± 2 °C with agitation (80 rpm) in a constant temperature incubator. Experiments were performed in triplicates. The fungal growth medium adapted from Sheramata and Hawari [16] was slightly modified by replacing the yeast extract with nitrogenenous compounds and consisted of: 2 g/L KH_2_PO_4_, 0.7 g/L MgSO_4_, 2.5 mg/L thiamine hydrochloride, 0.14 g/L CaCl_2_ · 2H_2_O, 0.07 g/L FeSO_4_ · 7H_2_O, 0.046 g/L ZnSO_4_ · 7H_2_O, 0.035 g/L MnSO_4_ · H_2_O, 0.007 g/L CuSO_4_ · 5H_2_O, 2.3 g/L disodium tartrate, 0.067 g/L veratryl alcohol, 0.5 g/L soybean phospholipids, and 0.1, 0.2 or 1 g/L ammonium sulfate (NH_4_)_2_SO_4_ or 1 g/L yeast extract as the nitrogen source, pH 5.0. In a typical experiment, a 40-mL glass vial was filled with 4.8 mL of growth medium, and 0.1 mL of appropriate glycerol stock solutions to yield final concentrations of 0.5, 1 or 10 g/L. The mixture was then autoclaved at 120 °C for 20 minutes. Following sterilization, the microcosms were inoculated with a *Phanerochaete chrysosporium* spore suspension (0.1 mL). CL-20 was then added to the vials from appropriate stock solutions made in acetone (25 μL) to attain the desired initial concentration (1, 3, 7, 100 or 500 mg/L). To the sterile controls, 0.1 mL of autoclaved growth medium was added instead of inoculum. The total reaction volume was 5 mL with an inoculum ratio of 1:50 (by volume). The inoculated microcosms contained approximately 2x10^7^ spores/mL as determined by plate counts. For time course studies, CL-20 concentration was monitored by mixing the whole degradation mixture with acetonitrile (1:1, v/v) and withdrawing 1 mL samples to be analyzed immediately by HPLC. Fungal growth was estimated gravimetrically as dry biomass, obtained by drying the separated fungal pellets at 105 °C overnight.

Some vials (microcosms) were supplemented with uniformly labeled [^14^C]-CL-20 (109.5 nCi) to yield a concentration of 7 mg/L and fitted with a small test tube containing 1 mL of 0.5 N NaOH, to trap the liberated ^14^CO_2_. These vials were sealed with PTFE Mininert screw-cap valves to prevent the loss of ^14^CO_2_. NaOH trap solution was routinely sampled and replaced through the septum of the screw-cap valves. To maintain aerobic conditions, 20 mL of the headspace air was replaced with the same amount of filtered (0.2 μm) fresh air, after each sampling event.

## 3. Results and Discussion

### 3.1. Treatment of CL-20 and its Hydrolysates with Aerobic Sludge

#### 3.1.1. Direct Treatment of CL-20 with Activated Sludge

Under all the experimental conditions tested, no significant CL-20 degradation relative to the sterile controls was observed over a period of 16 weeks. Some minor CL-20 depletions observed in both the control and experimental vials may be attributed to abiotic transformations, most likely hydrolysis, since the pH of the reaction medium was in the alkaline range at the end of the incubation period. This interpretation is supported by CL-20 hydrolysis work performed in our laboratory [25] and literature data [26] which indicate that CL-20 hydrolyzes at pH’s above neutral. On this basis, it was concluded that activated sludge process was not a viable option for the treatment of CL-20 containing wastewaters.

#### 3.1.2. Treatment of Radiolabeled [^14^C]-CL-20 Hydrolysates with Aerobic Sludge

**Figure 2.**
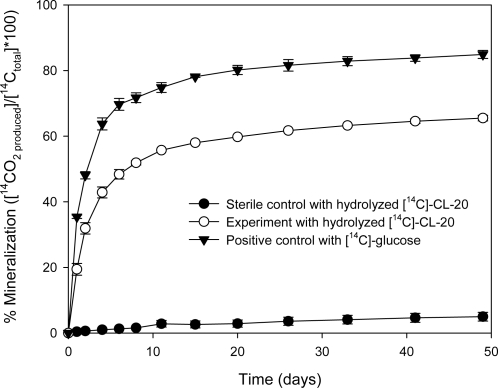
Mineralization of the hydrolyzed [^14^C]-CL-20 by activated sludge.

As shown in Figure 2, mineralization began shortly after incubation and 19.4% of the hydrolyzed [^14^C]-CL-20 was transformed into ^14^CO_2_ within 1 day. Within 11 days of incubation, the mineralization of the hydrolyzed [^14^C]-CL-20 accounted for 56% of the initial amount of [^14^C]-CL-20. Thereafter, the ^14^CO_2_ production rate decreased gradually and the extent of mineralization after 49 days reached a plateau of 65.5%. In the positive controls, the extent of glucose mineralization was 84.9% within the same time period, indicating that the microorganisms in the inocula were sufficiently active. The remaining carbon (15.1%) was possibly converted into biomass during incubation. The negative controls, which were supplemented with [^14^C]-CL-20 and sterilized medium have shown only 5% mineralization. At the end of the mineralization experiments, the microcosms were sacrificed in order to establish a [^14^C] material balance. The residual [^14^C] in the microcosms was 33.8% of the initially added radioactivity, as determined by sampling the aqueous fraction (5 mL) and analyzing by liquid scintillation counting. When this is combined with the [^14^C] recovered as ^14^CO_2_, a total ^14^C recovery of 99.4 % is obtained. It should be noted that the nearly complete [^14^C] recovery indicates that ^14^CO_2_ is not a by-product of the CL-20 alkaline hydrolysis reaction. These results suggest that alkaline hydrolysis coupled with aerobic biological treatment is a viable option for the destruction of CL-20 and its hydrolysis by-products.

### 3.2. Biotransformation of CL-20 by Phanerochaete Chrysosporium

The biodegradation studies using Phanerochaete chrysosporium involved two distinct approaches: (i) addition of CL-20 to the cultures during inoculation, and (ii) addition of CL-20 to pre-grown cultures of the fungus.

#### 3.2.1. Biodegradation Experiments with Growing Cultures of *Phanerochaete chrysosporium*

##### Effect of the Initial CL-20 Concentration on Biodegradation

**Figure 3.**
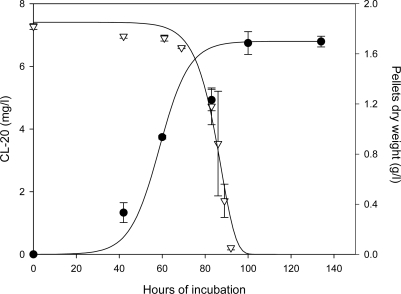
Time course profile for the degradation of CL-20 and biomass build-up in growing cultures of Phanerochaete chrysosporium with 10 g/L glycerol and 0.2 g/L ammonium sulfate at an initial CL-20 concentration of 7 mg/L; (•) pellets dry weight, (∇) CL-20 concentration, (**—**) logistic model (Equations 5 and 6).

**Figure 4.**
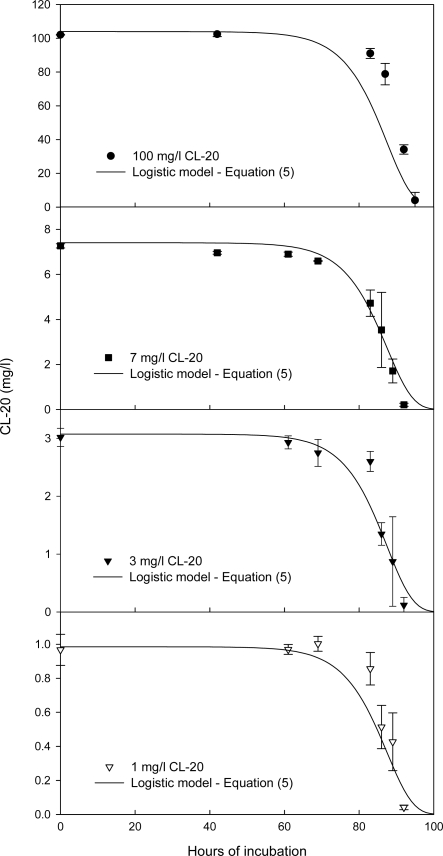
Time course profile for the degradation of CL-20 in growing cultures of *Phanerochaete chrysosporium* with 10 g/L glycerol and 0.2 g/L ammonium sulfate at initial CL-20 concentrations of 1, 3, 7 and 100 mg/L; data were fitted to the logistic model (Equation 5).

Figure 3 shows the time concentration profiles of CL-20 and biomass in growing culture of *Phanerochaete chrysosporium* for a CL-20 initial concentration of 7 mg/L. Substrate disappearance for other initial CL-20 concentrations (1, 3, 100 mg/L) followed virtually the same pattern as shown in Figure 4. The CL-20 concentration remained stable in non-inoculated controls. In all experiments Cl-20 biodegradation initiated after a lag phase of about 65 hours and was nearly fully transformed in less than 95 hours of incubation irrespectively of initial concentration, However, in microcosms containing 500 mg/L of initial concentration, CL-20 depletion did not exceed 10.4 ± 6.8% at an incubation period of 113 hours.

Dry biomass data presented in Table 1 show that the presence of CL-20, up to levels of 500 mg/L, did not adversely affect the biomass production in microcosms that contained varying initial concentrations of the nitramine. This finding suggests that CL-20 does not inhibit the growth of *Phanerochaete chrysosporium* within the concentration range studied. Similar findings have been reported in RDX studies where no noticeable differences in the produced mycelial mass for RDX levels up to 250 mg/L [27]. In contrast to CL-20, however, *Phanerochaete chrysosporium* was not able to biodegrade RDX above its solubility limit (approximately 80 mg/L). Therefore, CL-20 appears to be more amenable to biodegradation by the fungus, compared to RDX.

##### Kinetics of Fungal Growth and CL-20 Degradation

The logistic model [28], employed successfully in fungal systems [29], was used to fit the experimental data (Figure 3).

In its differential form, the logistic equation is presented as follows:

(1) $$\frac{dX}{dt}\;=\;r.X\;\left(1-\frac{X}{X_{\text{max}}}\right)$$

Equation (1) can be solved to obtain:

(2) $$X\;=\;\frac{X_{\text{max}}}{1+\left(\frac{X_{\text{max}}-X_{o}}{X_{o}}\right)\;{\text{exp}}^{-rt}}$$

where r is the maximum specific growth rate in a particular environment, X is the population density, X_0_ is the initial population density, X_max_ is the maximum population density achievable in that environment, and t is time. The substrate disappearance rate is related to biomass growth by:

(3) $$\frac{dS}{dt}\;=\;-\frac{k\cdot S\cdot X_{\text{max}}}{1+\left(\frac{X_{\text{max}}-X_{o}}{X_{o}}\right)\;{\text{exp}}^{-rt}}$$

where S is the substrate concentration.

Integration of the equation (3) yields:

(4) $$S\;=\;S_{o}{\left[\varphi ({\text{exp}}^{r\cdot t}-1)+1\right]}^{-\text{k}/\text{r}}$$

where $\varphi \;=\;\frac{X_{o}}{X_{\text{max}}}$ and $k\;=\;\frac{V_{\text{max}}\;X_{\text{max}}}{K_{m}}$

V_max_ is the maximum specific reaction rate and K_m_ is the half-saturation constant.

Elapsed time, t in Equation (4) was adjusted by subtracting the observed lag times, t_L_, to account for the period during which no noticeable CL-20 utilization occurs. A regression analysis was used to derive the kinetic parameters in Equations (2) and (4) using CL-20 and biomass concentration data obtained from experiments conducted at an initial energetic concentration of 7 mg/L with 10 g/L glycerol and 0.2 g/L ammonium sulfate with initial and maximum biomass concentrations of 0.00034 g/L and 1.7 g/L, respectively. The values of k and r are obtained as 13.5 h^−1^ and 0.145 h^−1^, respectively. Using the obtained parameters, a general model for CL-20 biodegradation could be written as:

(5) $$S\;=\;S_{0}{\left[\frac{0.00034}{X_{\text{max}}}\;({\text{exp}}^{0.145\;t}-1)+1\right]}^{-93.1}$$

(6) $$X\;=\;\frac{X_{\text{max}}}{1+\left(\frac{X_{\text{max}}-0.00034}{0.00034}\right)\;{\text{exp}}^{-0.145\;\;\;t}}$$

Figure 4 shows that the logistic growth kinetic model (Equation 5) fits well the CL-20 biodegradation time concentration profiles over a wide range of initial CL-20 concentrations (1–100 mg/L).

##### Effects of Nutrient Types and Levels on Biodegradation

**Figure 5.**
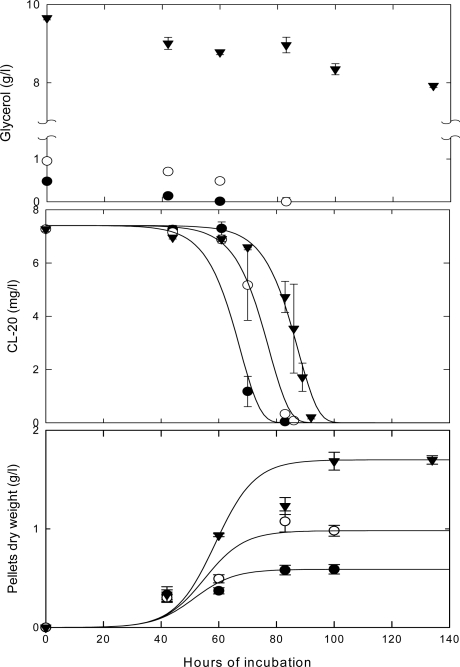
Time course profile for: (a) carbon source (glycerol) consumption, (b) removal of CL-20, and (c) biomass build-up in growing cultures of *Phanerochaete chrysosporium* incubated at low C-low N: (•) 0.5 g/L or (○) 1 g/L glycerol and 0.2 g/L of ammonium sulfate; and high C-low N: (▾) 10 g/L glycerol and 0.2 g/L of ammonium sulfate (**—**) logistic model (Equations 5 and 6).

**Figure 6.**
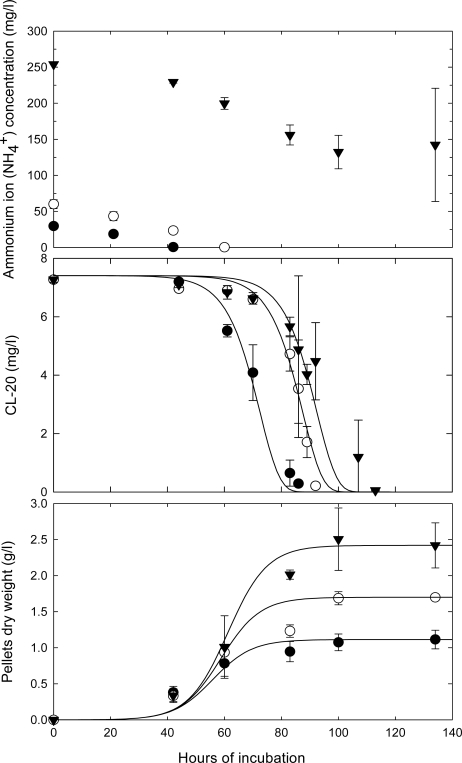
Time course profile for the biomass build-up, nitrogen source (ammonium sulfate) consumption and removal of CL-20, in growing cultures of *Phanerochaete chrysosporium* incubated at high C-low N: 10 g/L glycerol and (•) 0.1 g/L or (○) 0.2 g/L of ammonium sulfate; and high C-high N: (▾) 10 g/L glycerol and 1 g/L of ammonium sulfate (**—**) logistic model (Equations 5 and 6).

**Figure 7.**
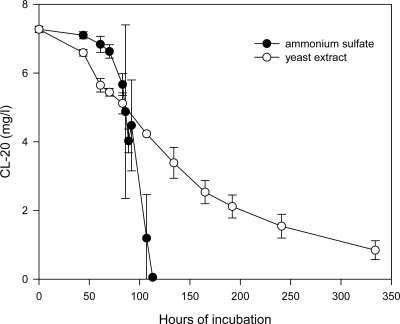
Time course profile for the degradation of CL-20 in growing cultures of *Phanerochaete chrysosporium* incubated in high C-high N media (10 g/L glycerol and 1 g/L ammonium sulfate or yeast extract).

The various carbon to nitrogen (C:N) ratios used in the fungal experiments are shown in Table 2. The experimental results are shown in Figures 5, 6 and 7.

The results presented in Figures 5a,b show that the CL-20 degradation by growing cultures of *Phanerochaete chrysosporium* does not require carbon starvation. The explosive was almost fully degraded in low C and high C media, however, longer lag phases were observed at higher initial glycerol levels. Biodegradation in low C media did not appear to initiate in response to carbon limitation. At the 0.5 or 1 g/L levels, glycerol was below detectable limits within 60 and 83 hours of incubation, respectively. At the 10 g/L level, 20% of glycerol was consumed, almost linearly, within 134 hours. The corresponding growth curves (Figure 5c) indicate that providing higher amounts of glycerol results in substantially higher fungal biomass growth.

The effect of nutrient nitrogen concentration on CL-20 disappearance at initial concentrations of glycerol (10 g/L) and CL-20 (7 mg/L) is shown in Figure 6. CL-20 was consumed simultaneously with ammonium sulfate in growing cultures of the fungus in high N medium (1 g/L ammonium sulfate). In low N media (0.1 g/L and 0.2 g/L ammonium sulfate), degradation occurred as soon as the nitrogen was exhausted. In the latter case, secondary metabolism was probably initiated in response to limitation of nitrogen.

Approximately 50% of ammonium sulfate was utilized in cultures that contained 1 g/L of the chemical. It can be concluded that CL-20 biodegradation by *Phanerochaete chrysosporium* does not necessarily require nitrogen limitation; however the process is delayed as the amount of the nitrogen in the medium is increased. Conversely, as shown in Figure 6, increasing the concentration of ammonium sulfate in the nutrient medium significantly enhanced the growth of the fungi.

While the general observation is that the optimal production of the ligninolytic enzymes requires nutrient limitation, there are many studies reporting biodegradation of pollutants under both limited and rich nitrogen and/or carbon conditions. The results indicate that, the amounts of supplied nutrient nitrogen and carbon are important factors that control the CL-20 biodegradation in growing cultures of *Phanerochaete chrysosporium*. The absence of nitrogen limitation requirement may be attributed to the possible dependence of CL-20 degradation on the MnP enzyme, rather than LiP; since MnP can be produced in nitrogen sufficient media [30]. This is supported by the findings of Fournier *et al.* [9], which indicated direct degradation of CL-20 by MnP. The principal role of Mn^2+^ dependent peroxidase is related to oxidation of Mn^2+^ to Mn^3+^, which then binds to an appropriate ligand, which in turn becomes a strong oxidant and diffuses into the substrate structure. Although the precise role and mechanism of MnPs in degradation of secondary metabolites is not clear, high levels of these lignonoytic enzymes usually correspond to the ability of white rot fungi to attack the nitrated energetic molecules. Our results indicate that cells supplemented with high nitrogen attack a CL-20 for more nitrogen, although their normal physiology is to degrade large molecules when they are nitrogen starved. This could be explained by the work of Kapich *et al*. [31]. They observed very low MnP activity in the absence of peptone whereas the addition of this organic nitrogen source increased considerably the activity of MnP. They reported that high concentration of organic nitrogen (up to 3–4 g/L of peptone) did not repress MnP production in P. chrysosporium but instead stimulated it.

On the other hand, Stahl *et al.* [27] reported that the amount of RDX degraded by growing *Phanerochaete chrysosporium* cultures was approximately 10 times higher under nitrogen-limited conditions as compared to nitrogen-sufficient (non-ligninolytic) conditions. They also reported that RDX was directly amenable to degradation by MnP, but not LiP [25].

The effect of the type nitrogen source (organic vs. inorganic) on the CL-20 biotransformation is shown in Figure 7. Ammonium sulfate and yeast extract (1 g/L) were selected to represent the two different nitrogen sources. The yeast extract, having a lower nitrogen content (about 10%) than ammonium sulfate (21%), was supplied at the maximum nitrogen requirement of the fungus for the given conditions, based on the data obtained from experiments with ammonium sulfate. The use of yeast extract resulted in comparable maximum biomass production (measured as 2.14 ± 0.16 g/L pellets dry weight at 134 hours of incubation) but significantly slower disappearance of CL-20 in the growing cultures, in comparison to ammonium sulfate (Figure 7). Both nitrogen sources resulted in comparable initial biotransformation rates, however, after 14 days of incubation, 11.6% of the initial CL-20 (7 mg/L) still remained in microcosms containing yeast extract. The slower biodegradation rates observed when using yeast extract, as the nitrogen source, may be attributed to the possible alteration of the ligninolytic enzyme system of the fungus. Sensitivity of these enzymes to various nitrogenous compounds has been reported in the literature. For example, Vahabzadeh *et al.* [32] reported that when urea was used as a nitrogen source, decolorizing ability of *Phanerochaete chrysosporium* was affected significantly and no MnP or LiP activities were detected. Also, non-ligninolytic conditions were observed using malt extract in the nutrient medium [33].

The potential ability of *Phanerochaete chrysosporium* to obtain its nutrient nitrogen from CL-20 was also investigated. When exposed to CL-20 (500 mg/L) as the sole nitrogen source in the presence of external carbon (glycerol), the fungus was able to grow, but at extremely slow rates with little pellet growth, compared to cultures containing supplemental nitrogen. While no CL-20 depletion or pellet formation was detected up to 22 days, at 35 days 30 ±3% of the nitramine was consumed by the microorganism. This indicates that *Phanerochaete chrysosporium* may utilize CL-20 as the sole nitrogen source in nitrogen-deficient environments. However, providing an external nitrogen source greatly enhances the rates of fungal growth and CL-20 disappearance.

##### Mineralization of [^14^C]-CL-20 by Phanerochaete Chrysosporium

**Figure 8.**
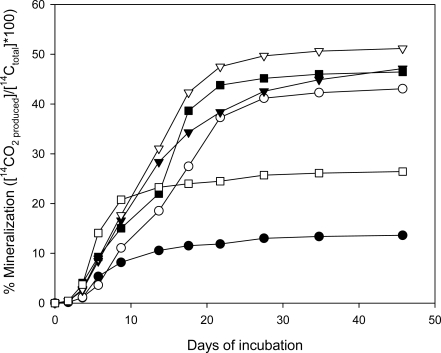
Mineralization of CL-20 by growing cultures of *Phanerochaete chrysosporium* incubated at various nutrient conditions: (▪) high C-high N: 10 g/L glycerol and 1 g/L yeast extract, (∇) high C-high N: 10 g/L glycerol and 1 g/L ammonium sulfate, (▾) high C-low N: 10 g/L glycerol and 0.2 g/L ammonium sulfate, (○) high C-low N: 10 g/L glycerol and 0.1 g/L ammonium sulfate, (□) low C-low N: 1 g/L glycerol and 0.2 g/L ammonium sulfate, (•) non-inoculated control.

The experimental results of CL-20 mineralization ability of *Phanerochaete chrysosporium* using uniformly labeled [^14^C]-CL-20 are presented in Figure 8. CL-20 mineralization began after 2 days of incubation in all vials. The explosive was depleted in less than 100 hours after inoculation in high C-low N medium, however, the radioactivity count ratio of the liberated ^14^CO_2_ to the total initial [^14^C]-CL-20 did not exceed 8.5% within this period. When the cultures were incubated for 46 days, considerable mineralization of CL-20, accounting for 47.1% of the initial [^14^C]-CL-20 (7 mg/L) was observed. In high C-low N medium, the extent of mineralization observed was 42.9%. When the ammonium sulfate concentration increased to 1 g/L (high C-high N), mineralization was slightly higher (51.2%), within the same incubation period. In general, the cumulative amount of liberated ^14^CO_2_ was similar at all nitrogen levels and did not significantly change after 28 days of incubation (Figure 8).

Incubation at high N–high C conditions using 1 g/L yeast extract and 10 g/L glycerol resulted in a mineralization level of 46.4%. Despite the differences in transformation rates, comparable mineralization was observed using yeast extract and ammonium sulfate. This suggests that, different enzymatic systems might play role in biotransformation and subsequent mineralization of the metabolites.

On the other hand, in low C-low N medium (1 g/L glycerol and 0.2 g/L ammonium sulfate), only 26.4% mineralization was detected within 46 days (Figure 8). Poor mineralization at low C conditions cannot be attributed to the comparatively little pellet growth (Figure 5), since high C-low N medium (10 g/L glycerol and 0.1 g/L ammonium sulfate) yielded similar biomass levels (Figure 6) but considerably higher ^14^CO_2_ production. Moreover, mineralization of CL-20 may not be dependent on the lignin degrading enzymes (i.e., MnP), in view of the results obtained by Fournier *et al.* [9] indicating that the main CL-20 biodegradation product glyoxal is not amenable to mineralization in purified enzyme cultures. Therefore, it may be concluded that presence of excess carbon is necessary to effectively mineralize the CL-20 metabolites by the growing fungus; although transformation of the nitramine appears to be more rapid at low carbon conditions (Figure 5).

During the incubation period (46 days), about 10% mineralization was detected in sterile non-inoculated controls, possibly due to abiotic transformations. The residual [^14^C] in the microcosms was determined by transferring the contents of the microcosms into a scintillation vial and analyzing by liquid scintillation counting. When combined with the [^14^C] recovered as ^14^CO_2_, total ^14^C recoveries in the range of 90.5 – 102.1% were obtained.

Using growing cultures of *Phanerochaete chrysosporium*, Fournier *et al.* [9] reported a considerably higher (80%) maximum mineralization of CL-20, in a nitrogen-limited growth medium containing 1.2 mM ammonium tartrate. Besides the nitrogen source, different incubation conditions, use of glycerol instead of glucose (10 g/L) as a carbon source and/or higher initial CL-20 in our medium (10-fold) may be responsible for the higher mineralization reported by Fournier *et al.* [9].

#### 3.2.2. Biodegradation Experiments with Pre-Grown Cultures of *Phanerochaete chrysosporium*

##### Roles of Mycelial Mass and Extracellular Fluid in Biodegradation

**Figure 9.**
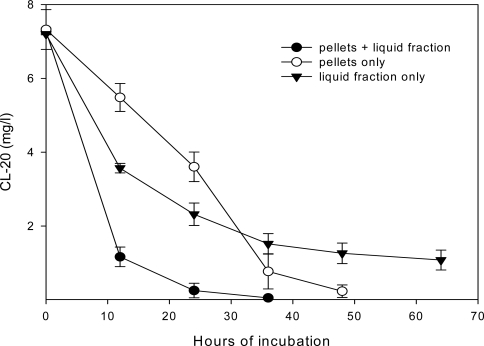
CL-20 disappearance profile in nitrogen-limited grown (100-hours old) cultures of *Phanerochaete chrysosporium*, using liquid fraction and pellets only, or both.

In an attempt to determine the impacts of extracellular enzymes, CL-20 was separately exposed to the liquid supernatant and the pellet fractions of *Phanerochaete chrysosporium*. Figure 9 presents the CL-20 degradation profile in cultures containing liquid fraction or pellets only, or both.

The biodegradation of CL-20 started without any lag phase, in all cases. Microcosms containing both pellets and liquid fraction rapidly transformed the CL-20 within 36 hours. Also, nearly complete degradation of CL-20 was observed when using only cell mass (without extracellular enzymes), within 48 hours. However, the initial rate of CL-20 disappearance was considerably less than the one observed using both extracellular enzymes and biomass, indicating extracellular enzymatic mediated reaction. Transformation rate increased after 24 hours, which indicates that extracellular enzymes were restored in the system. In cultures containing only the liquid fraction, degradation occurred, but slowed remarkably after the first 24 hours and reached a plateau, after an 85% CL-20 depletion. This may suggest a loss of enzymatic activity over time.

##### Effect of Nutrient Levels on Biodegradation

**Figure 10.**
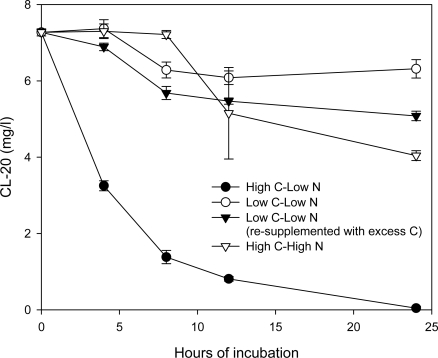
CL-20 disappearance profile in 6 days-old *Phanerochaete chrysosporium* cultures with different nutrient C:N ratios (•) high C-low N: 10 g/L glycerol and 0.2 g/L ammonium sulfate, (○) low C-low N: 1 g/L glycerol and 0.2 g/L ammonium sulfate, (▾) low C-low N: 1 g/L glycerol and 0.2 g/L ammonium sulfate (re-supplemented with 10 g/L glycerol during CL-20 addition), (∇) high C-high N: 10 g/L glycerol and 1 g/L ammonium sulfate.

CL-20 transforming ability of pre-grown cultures of *Phanerochaete chrysosporium*, at various nutrient conditions was investigated. Figure 10 shows the CL-20 disappearance profile in 6 days-old cultures, grown with different nutrient C:N ratios: high C-low N (10 g/L glycerol and 0.2 g/L ammonium sulfate), low C-low N (1 g/L glycerol and 0.2 g/L ammonium sulfate), and high C-high N (10 g/L glycerol and 1 g/L ammonium sulfate). During CL-20 addition, glycerol levels were replenished in several microcosms which contained low C-low N growth medium, in order to bring the concentration of the substrate to excess levels.

Within 24 hours, CL-20 (7 mg/L) was completely transformed in cultures with high C- low N, however; only 13% of it was degraded at low C-low N conditions, i.e. when glycerol was initially supplied at 1 g/L. In the latter case, the degradation took place mostly up to 8 hours following CL-20 addition, and the remaining CL-20 was more or less the same level at 8 and 24 hours. Replenishing the glycerol level resulted in only little improvement in *Phanerochaete chrysosporium* degradation ability, achieving 30% biodegradation, within the same period. An excess of ammonium sulfate (high N-high C) suppressed degradation significantly and 56% of the initially added CL-20 remained in the reaction medium 24 hours after the addition of the nitramine. Assuming that MnP is the major degrading enzyme for CL-20, this result is not surprising according to several studies that report maximum MnP activities under nitrogen-limited conditions, in *Phanerochaete chrysosporium* cultures [34,35].

In conclusion, 6-days old grown cultures of *Phanerochaete chrysosporium* were effective in biotransformation of CL-20 in growth media containing excess carbon, with depleted nitrogen. This indicates that the continuous activity of the CL-20 transforming mechanism is best maintained at the aforementioned conditions.

##### Effect of Culture Age on Biodegradation

Operation of CL-20 remediation systems with *Phanerochaete chrysosporium*, such as biofilters, rely on the biodegradation ability of the fungal pellets for extended periods, depending on maintenance of high levels of enzymatic activity. Pelleted growth form of the white rot fungi offers the possibility of biomass reuse and thereby continuous operation of such processes. In order to evaluate the potential of aged *Phanerochaete chrysosporium* pellets to transform CL-20, the nitramine was added into cultures at various ages and its disappearance was monitored. Cultures were initially grown under high C-low N (10 g/L glycerol and 0.2 g/L ammonium sulfate) conditions.

**Figure 11.**
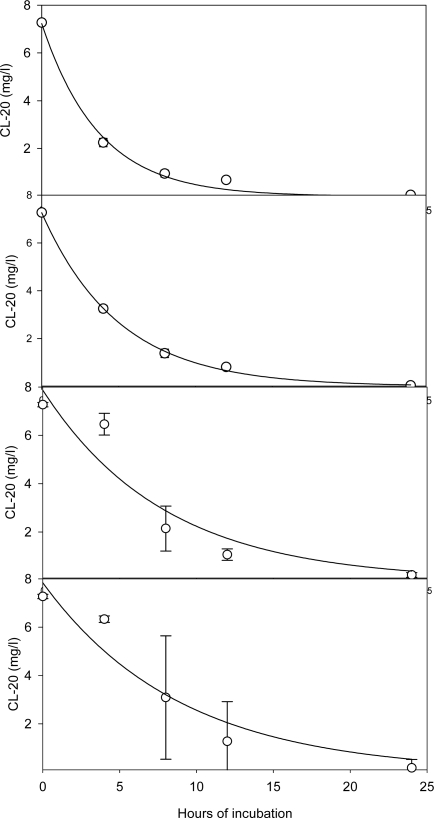
CL-20 degradation profile in nitrogen-limited pre-grown *Phanerochaete chrysosporium* cultures of various ages (a) 5-days (b) 6-days (c) 10-days (d) 18-days (Solid lines represent the non-linear regression curve fits, assuming pseudo-first order kinetics).

Aged cultures, up to 18 days, were able to fully transform the newly added CL-20 (Figure 11), following pseudo-first order kinetics. Such kinetics for pre-grown cultures has previously been suggested to apply to white rot fungi co-metabolism [18]. First order degradation rate constants computed for 5, 6, 10 and 18-days aged cultures were 0.269 (0.023), 0.200 (0.006), 0.126 (0.038), and 0.112 (0.027) hour^−1^, respectively, with corresponding correlation coefficients of 0.9967, 0.9995, 0.9470 and 0.9628. The rate constants were determined using non-linear regression analysis. The values in parentheses are standard errors. A drop in biodegradation rate and deviation from pseudo-first order kinetics behavior are observed as the culture age increases from 6 to 10 days.

## 4. Conclusions

Activated sludge was found ineffective in transforming CL-20 either in the presence or absence of supplemental nitrogen and carbon sources. The moderate depletions observed could not be attributed to biodegradation, as the non-inoculated controls exhibited similar behavior, most likely of hydrolytic nature. However, activated sludge was able to mineralize the base hydrolysis products of CL-20 to carbon dioxide significantly. It can be concluded that alkaline hydrolysis coupled with aerobic microbial treatment is a technically feasible option for the removal of CL-20 and its by-products from contaminated waste streams and groundwater.

The white rot fungus *Phanerochaete chrysosporium* was capable of degrading CL-20 in the presence of supplementary carbon and nitrogen sources. The results show that external nitrogen and carbon sources are important factors controlling the CL-20 biodegradation in growing cultures. Pre-grown cultures of *Phanerochaete chrysosporium* were most effective in biotransformation of the nitramine in growth media containing excess carbon and limited nitrogen. *Phanerochaete chrysosporium* was able to degrade CL-20 up to concentrations of 100 mg/L. It was observed that CL-20 does not inhibit the growth of the fungus up to concentrations of 500 mg/L. CL-20 biodegradation by *Phanerochaete chrysosporium* was found to follow the logistic kinetic growth model. While readily transformed, CL-20 is only slowly mineralized in *Phanerochaete chrysosporium* cultures.

## Figures and Tables

**Table 1.** Dry biomass quantities in *Phanerochaete chrysosporium* cultures with varying initial CL-20 levels, at 100 hours of incubation.

**CL-20 concentration (mg/L)**	**Dry biomass (g/L)**
0	1.506 (0.211)*
1	1.704 (0.021)
3	1.534 (0.123)
7	1.556 (0.066)
100	1.569 (0.147)
500	1.574 (0.123)

* values in parentheses are standard deviations.

**Table 2.** Summary of the studied nutrient carbon and nitrogen combinations.

	**Glycerol (g/L)**	**Ammonium Sulfate (g/L)**	**Yeast Extract (g/L)**
low C-low N	0.5	0.2	
low C-low N	1	0.2	
high C-low N	10	0.2	
high C-low N	10	0.1	
high C-high N	10	1	
high C-high N	10		1
